# Human liver segments: role of cryptic liver lobes and vascular physiology in the development of liver veins and left-right asymmetry

**DOI:** 10.1038/s41598-017-16840-1

**Published:** 2017-12-07

**Authors:** Jill P. J. M. Hikspoors, Mathijs M. J. P. Peeters, Nutmethee Kruepunga, Hayelom K. Mekonen, Greet M. C. Mommen, S. Eleonore Köhler, Wouter H. Lamers

**Affiliations:** 1 Department of Anatomy & Embryology, Maastricht University, Maastricht, The Netherlands; 2 Department of Anatomy, Faculty of Science, Mahidol University, Rama VI Road, Bangkok, 10400 Thailand; 3 NUTRIM Research School of Nutrition and Translational Research in Metabolism, Maastricht, The Netherlands; 4 Tytgat Institute for Liver and Intestinal research, Academic Medical Center, Amsterdam, The Netherlands

## Abstract

Couinaud based his well-known subdivision of the liver into (surgical) segments on the branching order of portal veins and the location of hepatic veins. However, both segment boundaries and number remain controversial due to an incomplete understanding of the role of liver lobes and vascular physiology on hepatic venous development. Human embryonic livers (5–10 weeks of development) were visualized with Amira 3D-reconstruction and Cinema 4D-remodeling software. Starting at 5 weeks, the portal and umbilical veins sprouted portal-vein branches that, at 6.5 weeks, had been pruned to 3 main branches in the right hemi-liver, whereas all (>10) persisted in the left hemi-liver. The asymmetric branching pattern of the umbilical vein resembled that of a “distributing” vessel, whereas the more symmetric branching of the portal trunk resembled a “delivering” vessel. At 6 weeks, 3–4 main hepatic-vein outlets drained into the inferior caval vein, of which that draining the caudate lobe formed the intrahepatic portion of the caval vein. More peripherally, 5–6 major tributaries drained both dorsolateral regions and the left and right ventromedial regions, implying a “crypto-lobar” distribution. Lobar boundaries, even in non-lobated human livers, and functional vascular requirements account for the predictable topography and branching pattern of the liver veins, respectively.

## Introduction

The classical anatomy of the liver is well established, with left and right lobes separated by the falciform ligament on the diaphragmatic surface, and the round and venous ligaments on the visceral surface of the liver. The gallbladder, inferior caval vein and liver hilum further delineate the caudate and quadrate lobes. Because these external landmarks were not helpful in planning liver surgery, an anatomical description that was based on the architecture of the biliary and/or vascular trees was developed ~60 years ago^1–5^. All models use the branching pattern of either the portal and hepatic veins (“French” model^1–3^) or the bile ducts (“American” model^4,5^) as segmentation criteria, but differ in their nomenclature. Here we use the accepted “Brisbane 2000” nomenclature^6^, as modified by Bismuth (2013).

In all concepts, the liver is subdivided into right and left “hemi-livers” by a plane through the inferior caval and middle hepatic veins, the first bifurcation of the portal vein or bile duct, and the gallbladder (the Rex-Cantlie “line”)^1,3,4,7^. The plane through the inferior caval and right hepatic vein subdivides the right hemi-liver into 2 “sectors”, each being served by 2^nd^ order branches of the portal vein or bile duct. Hjorstjö, however, described the right hemi-liver to contain 3 sectors^5^, the extra sector arising from the symmetrically distributed 3^rd^ order branches of both bile duct and portal vein in the right anterior sector^5,8^. The sectors of the left hemi-liver differ between the French and American models, and are separated by a plane through the inferior caval vein on the one hand and the left hepatic vein^1^ or the umbilical recess/fissure^4^ on the other (Supplementary Figure 1). All sectors are subdivided into “segments” by a transversely oriented plane through the 1^st^ order branches of the portal vein^1^ or the distribution of the 3^rd^ order branches of the bile ducts^4^. The segments of both models are similar, except that segment 4 is divided into cranial and caudal portions in the American model only^4^. Couinaud’s segmental concept of the architecture of the liver^1^, as translated by Bismuth^9,10^, with 4 sectors and 8 segments, has become the prevailing standard in textbooks. Nevertheless, the concept remains controversial, because dissections and casts show that the “real” boundaries of its segments differ substantially from the “model” boundaries^11–14^. Furthermore, the claim that the right hemi-liver consists of 3^5^ rather than 2 sectors^1,3,4^ has resurfaced^8^, while the claim that the left hemi-liver consists of 3 segments^1,2,4^ is questioned because the main portal trunk may sprout as many as ~20 direct branches and, therefore, segments^14,15^. Moreover, the sector boundaries in the left hemi-liver differ in the French and American models ([1]vs [4]; *cf* ^16^;). A newer concept that is based on the distribution of the “main” branches of the portal vein and that acknowledges only 2 main hepatic veins^17,18^ sidesteps these issues and proposes a 4-lobe segmental model with a caudate lobe, 2 portal segments in right hemi-liver and 1 portal segment in the left hemi-liver. In all models, the caudate and quadrate lobes stand apart and don’t have well-defined boundaries.

Although the discrepancies between the models and the real liver can be solved in a practical manner by imaging of the liver vessels and deducing segmental boundaries from these images^19^, the conceptual issues appear to arise from a limited understanding of the branching pattern of the liver veins. In line, Majno *c*.*s*. and Bismuth recently called for a new anatomical foundation of the segmental architecture of the liver in this journal^20,21^. Because the main players invoke the embryology of the liver vessels to support their arguments^1,2,5,22–24^, we reasoned that a better understanding of the developmental appearance of the liver vessels and architecture would shed light on these issues and allow us to propose a consensus model.

In a study of early liver development^25^, we observed that both vitelline veins become incorporated into the two dorsolateral lobes or wings and both umbilical veins into the single ventromedial lobe of the liver, with the gallbladder marking the midline of the ventromedial lobe. In the 5^th^ week, portal veins started to branch off from the portal stem of the right vitelline vein, from the left umbilical vein, and from the portal sinus that connects both vessels in the liver hilum. In the present study, we followed the development of these early portal vessels and the appearance of the hepatic veins, and show that the definitive vascular configuration becomes established in the 7^th^ week. The branching pattern of the portal and hepatic veins appears to result from the blood-distributing function of the umbilical vein and the (crypto-)lobar architecture of the liver, respectively. We conclude that Couinaud’s model identifies surgically removable quantities of the liver rather than segments that reflect the branching order of the portal vein.

## Results

### Development of intra-hepatic portal venous system

#### Right-sided portal tree

**Figure 1:**
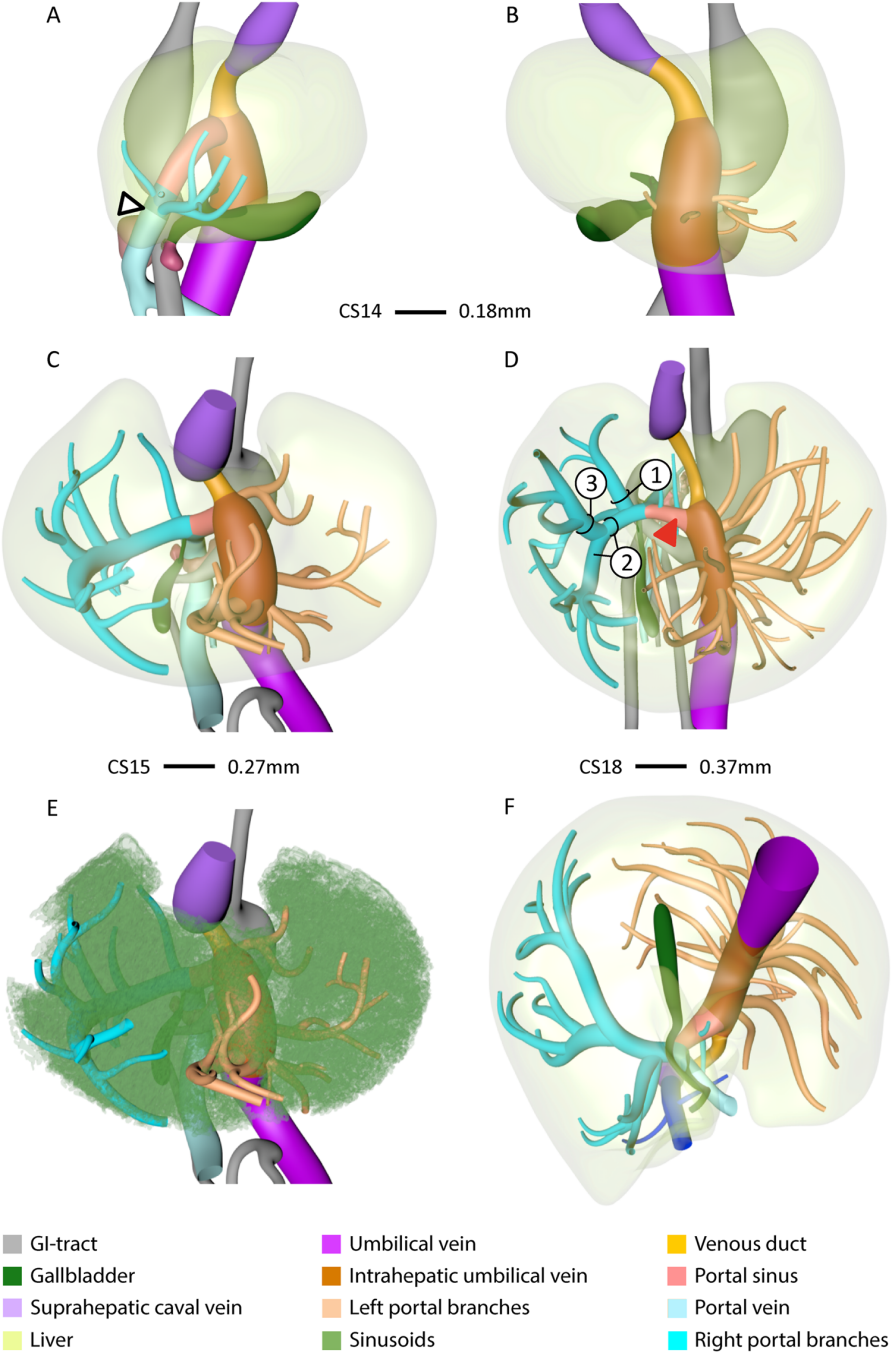
The appearance of portal veins in the liver. Right-sided (**A**), left-sided (**B**), ventro-cranial (**C**–**E**) and caudal (**F**) views of CS14 (**A**,**B**), CS15 (**C**,**E**), and CS18 (**D**,**F**) livers. Panel E is C with sinusoids retained. Blue and peach: portal veins in right and left hemi-livers, respectively. Between CS14 and CS18, the number of large portal branches was pruned to 3 in the right hemi-liver (**D**) in between the sinusoidal network (**E**), while >10 branches remained in the left hemi-liver. Note that portal veins do not cross Cantlie’s line (**F**). Arrowhead (**A**): entrance of portal vein into the liver.

The portal vein entered the liver on the right-side of the duodenum (arrowhead in Fig. 1A). From CS14 (34 days of development: Supplementary Fig. 1) onwards, branches from the intrahepatic stem of the portal vein (former right vitelline vein) extended into the liver periphery (blue vessels in Fig. 1), Initially, the number of separate twigs increased (Fig. 1A,C), but with progressing development, 3 branches began to predominate (Fig. 1D): branch #1 extended dorsolaterally, #2 ventrolaterally along the right outer edge of the liver, and branch #3 ventrocranially. Branch #3 was usually a side branch of #2. The branches supplied the posterior (#1), anterior (#2) and lateral (#3) portions of the right hemi-liver. Comparison of panels A, C and D underlines the continuing expansion of the vessels into the liver periphery with increasing age. Furthermore, higher order branches became apparent (Fig. 1C,D).

#### Left-sided portal tree

The portal twigs that branched off into the left hemi-liver (peach-colored vessels in Fig. 1B–F and Supplementary Fig. 2) arose from the intrahepatic portion of the left umbilical vein. The venous duct, a left-right shunt, connected the distal end of the umbilical vein in the liver hilum with the inferior caval vein. With time, more and more portal twigs appeared. At CS14, CS15, CS16, and CS18, we counted 4, 6, 6, and 12 such vessels. Between 6.5 (CS18) and 10 weeks, no further increase was observed. The position of the roots of these portal branches on the main umbilical trunk ascended in a spiraling fashion from anterior to lateral (Fig. 1D). Note that there are no branches on the posterior side of the intrahepatic umbilical vein (Fig. 1F), which reflects the establishment of Cantlie’s line (Fig. 1F and Supplementary Figure 2).

### Development of hepatic venous system

**Figure 2:**
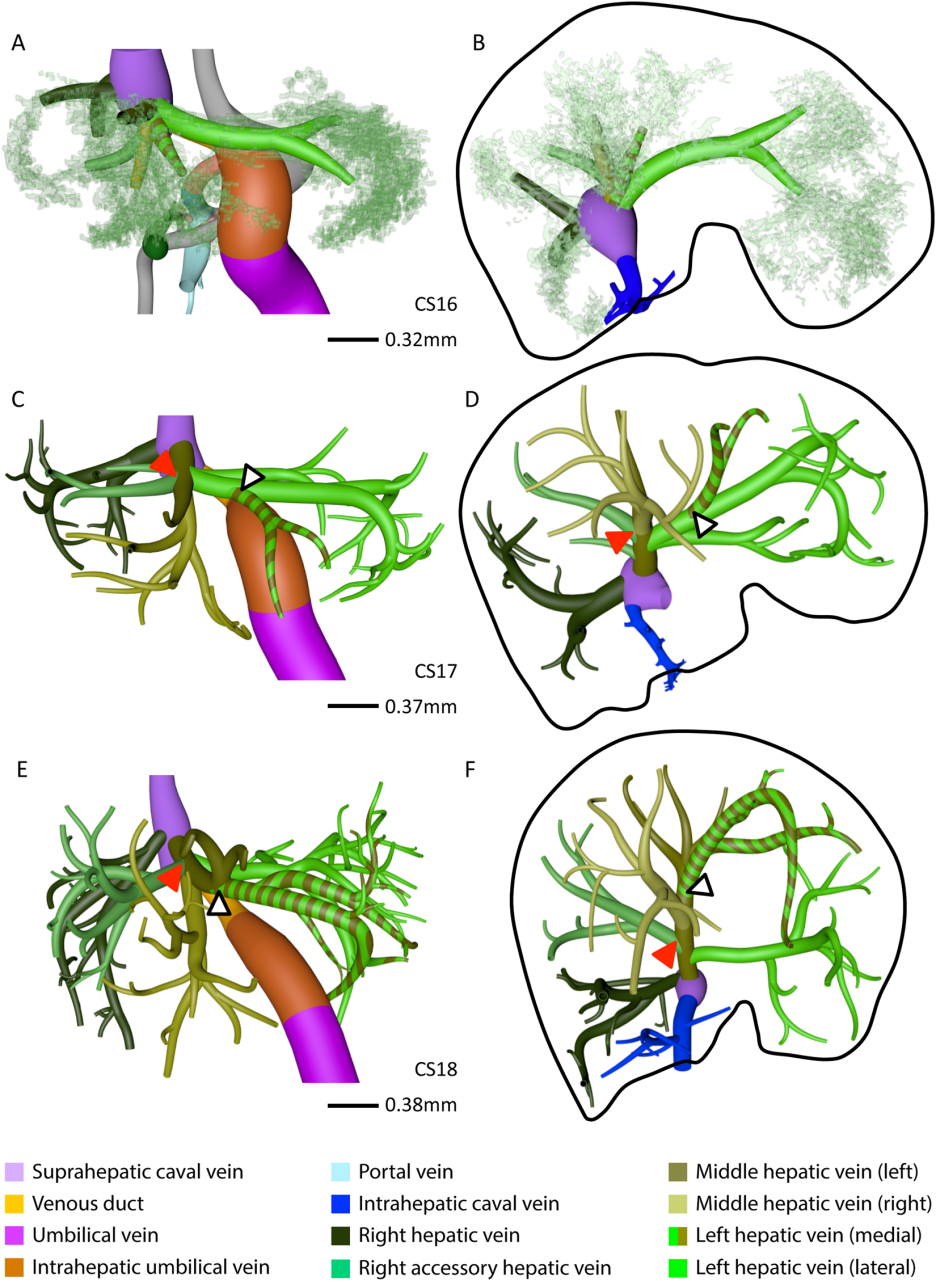
Hepatic vein development. Anterior (**A**,**C**,**E**) and caudal (**B**,**D**,**F**) views. Formation of 6 main hepatic veins, each identifiable by a different color, from sinusoidal network between 5.5 (CS16) and 6.5 (CS18) weeks of development. Red and open black arrowheads: variable course of right medial (accessory) and left medial hepatic veins, respectively; black line (**B**,**D**,**F**): liver contour.

**Figure 3:**
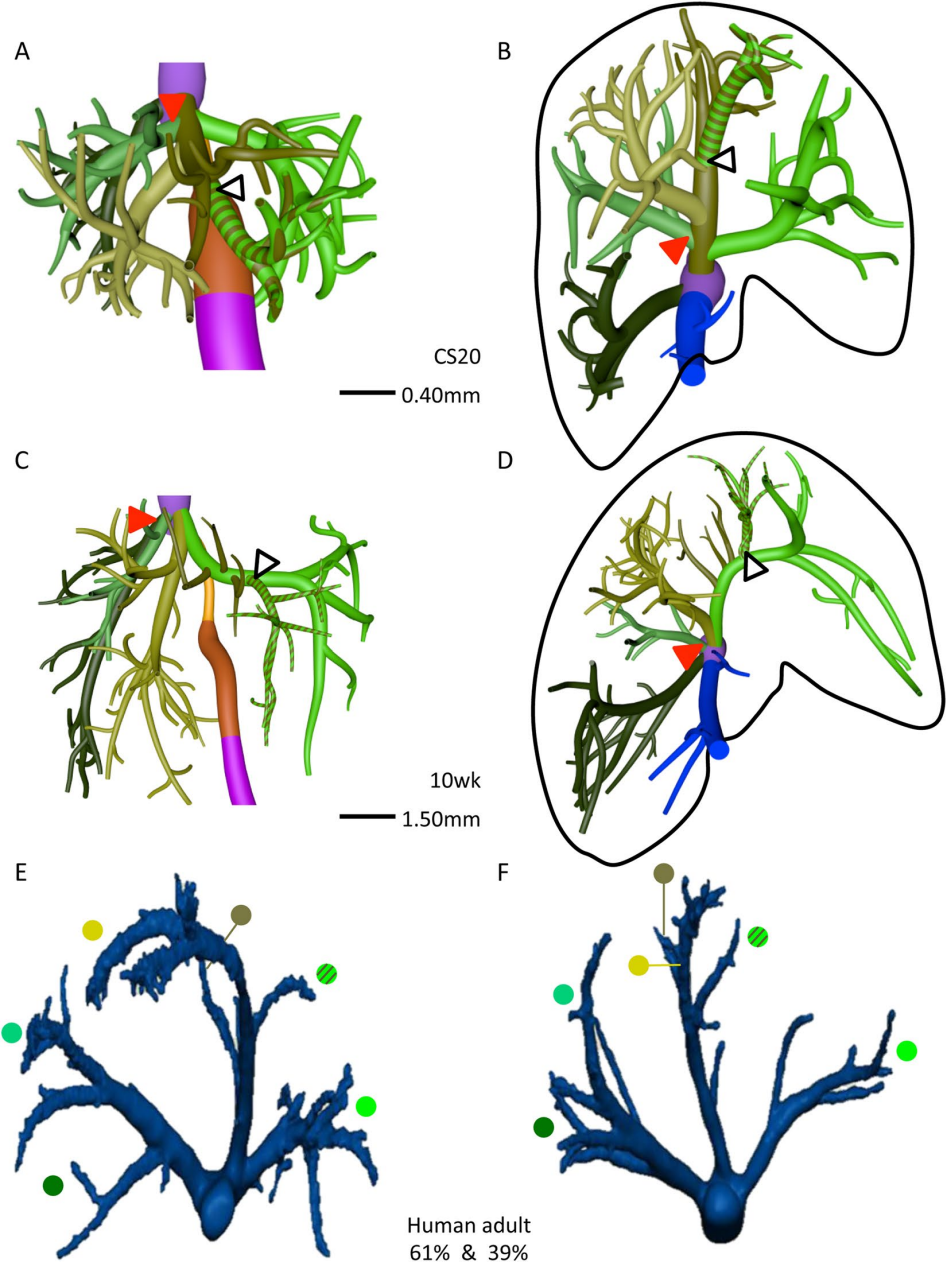
The distribution of the hepatic veins in embryonic and adult human livers. Anterior (**A**,**C**) and caudal (**B**,**D**–**F**) views. Panels A–D show the main branches of the hepatic veins in 7 and 10 week-embryos, while panels E,F show the hepatic veins as observed by Fang *c*.*s*. (^28^; reproduced with permission of the publisher). The color-coded dots in panels E,F identify the hepatic veins in adults that correspond with those in embryonic livers. Each hemi-liver contains 3 hepatovenous branches in both embryos and adults. Color code is identical to that of Fig. 2.

**Figure 4:**
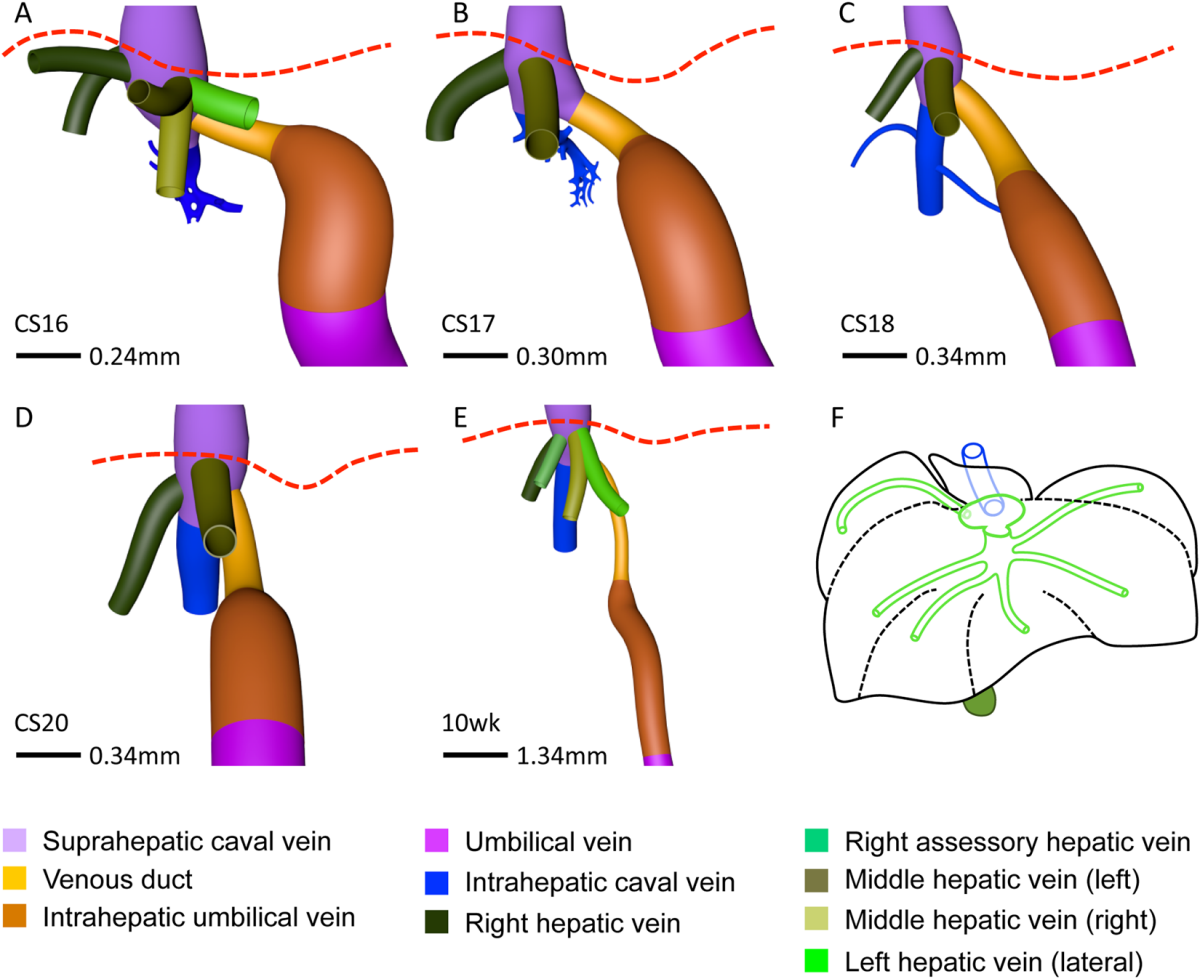
Various hepatic-vein configurations draining into the caval vein. Anterior (**A**–**E**) views. 3 or 4 hepatovenous trunks and venous duct drain directly into the suprahepatic portion of the inferior caval vein. The hepatic vein of the caudate lobe (blue) becomes the intrahepatic portion of the caval vein at CS18. Panel F shows the “prototype” of all 6 main hepatic veins. Red interrupted line: diaphragm.

As the portal veins expanded into the liver periphery, sinusoids in between them began to transform into hepatic veins (compare CS15 (Fig. 1E) with CS16 (Fig. 2A,B)). This process spread radially from the suprahepatic portion of the inferior caval vein, with 2 or 3 main hepatovenous trunks emptying into the caval vein (Figs 2–4). The right hepatic vein expanded caudally into the right dorsolateral side of the liver (Figs 2 and 3). The middle hepatic vein usually continued as 5 main tributaries: a right medial (often called accessory) hepatic vein in the lateral region of the right hemi-liver, a middle hepatic vein with left and right tributaries, and a left hepatic vein with left medial and left lateral tributaries (Figs 2,3 and 4F, and Supplementary Figure 2). The topography of the 5 main tributaries of the middle hepatic vein did not differ in the embryos we examined, but they reached the inferior caval vein either separately or after merging with a neighboring tributary. Two configurations were commonly observed: the left medial hepatic branch drained into the left lateral or the left tributary of the middle hepatic vein (open black arrowheads in Figs 2 and 3); the corresponding vessel on the right side, the right accessory hepatic vein, drained more proximally or distally into the middle hepatic vein (red arrowhead in Figs 2 and 3). In the 10-week embryo, for example, the middle and left hepatic veins shared a short common outflow into the inferior caval vein, while the right accessory hepatic vein drained directly into the caval vein (Fig. 3C,D). The distribution of hepatic veins in the human embryos (Figs 2 and 3) resembled that in adults (Fig. 3E,F), with 2 or 3 hepatic trunks draining directly into the inferior caval vein (Fig. 4), while the main tributaries were symmetrically divided over both hemi-livers (3 branches each; Fig. 4F). These findings show that the hepatovenous drainage pattern in human livers is established at CS17 or CS18.

#### Hepatic portion of inferior caval vein

The sinusoidal network in the developing caudate lobe formed a hepatic vein that joined the inferior caval vein on its posterior side separately from the other main hepatic veins. At CS18 (6.5 weeks) this vessel could be identified as the intrahepatic portion of the inferior caval vein (Fig. 2 and Supplementary Figure 2).

### Development of caudate and quadrate lobes

**Figure 5:**
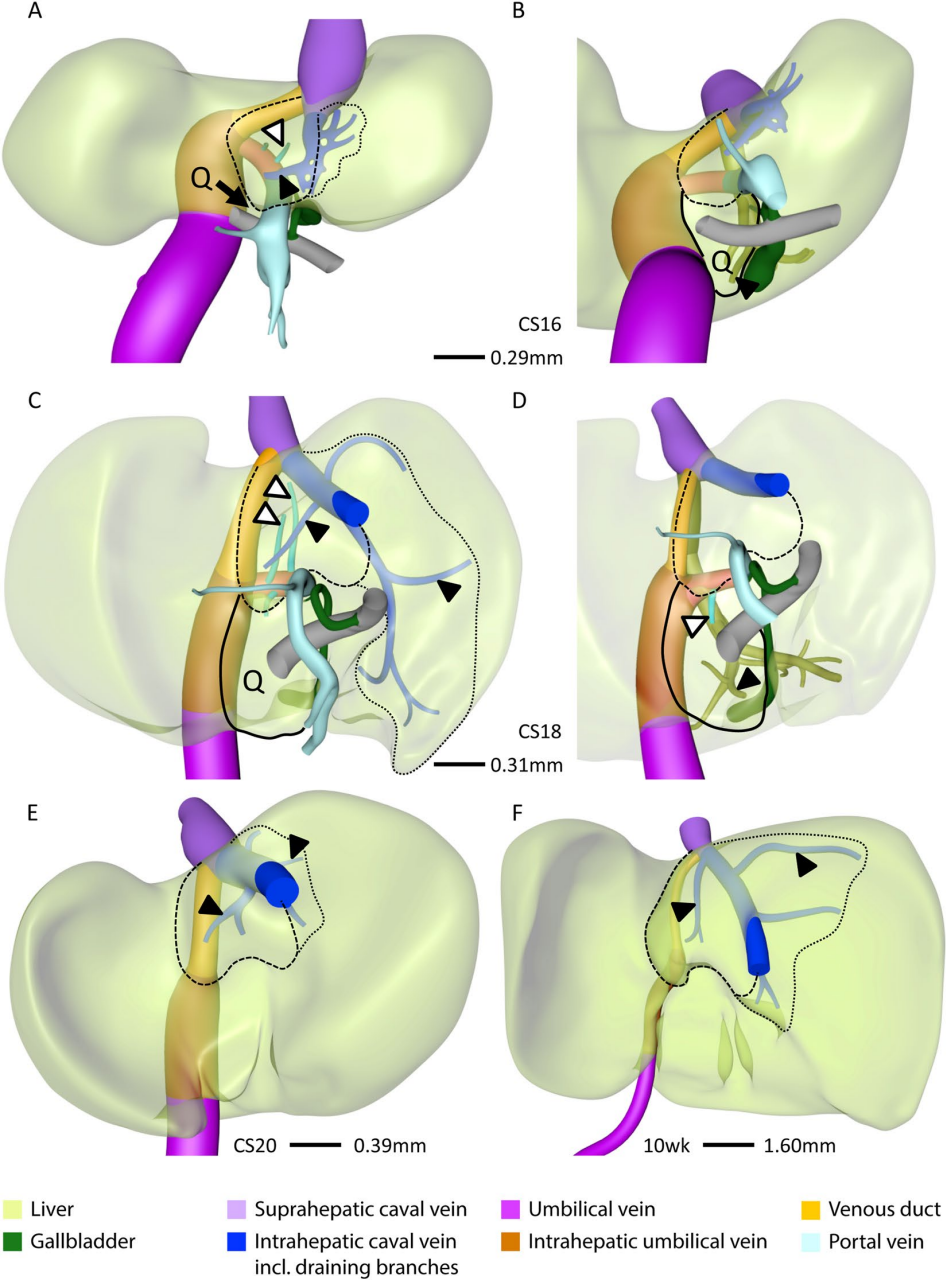
Development of the caudate and quadrate lobes. Posterior (**A**,**C**,**E**,**F**) and posterio-caudal (**B**,**D**) views at CS16 (**A**,**B**), CS18 (**C**,**D**), CS20 (**E**) and 10 weeks (**F**). The caudate lobe (interrupted line) appeared at CS16 and the quadrate lobe (Q; black line) at CS18, were supplied by branches from the portal sinus (black open arrowheads), and were drained by the intrahepatic caval vein and middle hepatic vein (black arrowheads), respectively. The number and length of hepatic-vein branches that drain unto the intrahepatic caval vein (blue) varies (dotted lines; A,C,E,F) and, therefore, the boundary of the caudate process is variable.

Concurrent with the development of the intrahepatic portion of the caval vein between CS16 and CS18 (5.5–6.5 weeks), the caudate and quadrate lobes became apparent on the posterior side of the liver (Fig. 5). The caudate lobe developed as a protrusion between the venous duct on its left side, the intrahepatic portion of the caval vein on its upper right side, and the portal sinus on its caudal side (Fig. 5A). The portal sinus is a left-right shunt between the distal end of the intrahepatic umbilical vein and the intrahepatic stem of the portal vein (red arrowhead in Fig. 1D) and will become the transverse part of the portal vein. From CS16 onwards, 2 small portal branches originating from the portal sinus supplied the caudate lobe (Fig. 5A–D; Supplementary Figure 2). Concurrent with the transformation of the hepatic vein of the caudate lobe into the intrahepatic portion of the inferior caval vein at CS18, small next-order hepatic veins became the draining vessels of the caudate lobe into the intrahepatic caval vein anteriorly and right laterally (Fig. 5C,E and F). There was no defined boundary between the caudate process and the right liver lobe in human livers, but based on the drainage area of hepatic veins^26^ the caudate process was variable in size among different human specimens we studied and could extend in some livers along the entire inferior part of the posterior sector of the right liver lobe (Fig. 5C,E and F).

**Figure 6:**
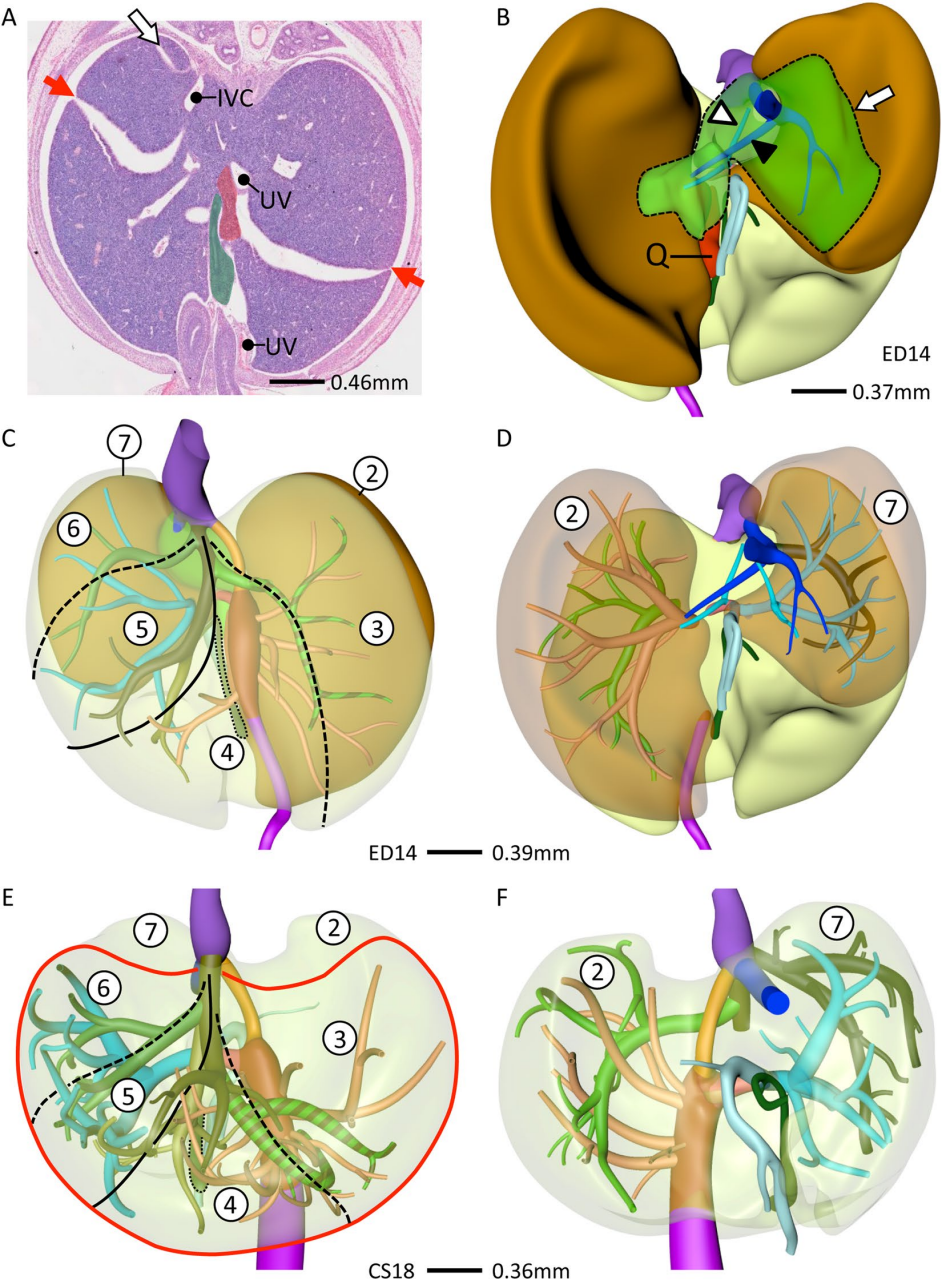
Lobar distribution of veins in human and mouse livers. Cranio-ventral (**C**,**E**) and posterior (**B**,**D**,**F**) views. In lobated livers, both caudate lobe and process (green) are free lobes that both drain into the intrahepatic caval vein (**B**). The murine quadrate lobe (Q, red) is small (**A**,**B**). The left and right dorsolateral lobes (B, brown) are drained by the left lateral and right hepatic veins (**D**). The ventromedial lobe (**E**, red line) is drained by 4 main hepatic branches (left and right medial, and the two upstream tributaries of the middle hepatic vein). The distribution of the hepatic veins is similar in mouse and human livers (*cf*. **B**–**D** and **E**,**F**). Using the lobar boundaries Couinaud’s surgical segments can be constructed: #1: caudate; #2 and #7: dorsolateral; and ##3–6: ventromedial with 3 hepatic veins. Symbols (**A**): red arrows: hepatic fissures; IVC: intrahepatic caval vein, UV: umbilical vein, green: gallbladder. Color code veins is as in Figs 1 and 2.

The quadrate lobe developed as a posterior protrusion from the left medial region of the liver between the intrahepatic umbilical vein (left-sided border), gallbladder (right-sided border) and portal sinus (cranial border) (Fig. 5B). It becomes identifiable at CS16 or CS17 (cf.^27^) and is anteriorly continuous with the liver sector between the umbilical fissure laterally and the gallbladder fossa medially. In agreement, the quadrate lobe became served by direct portal branches from the portal sinus at CS18 (Fig. 5D), while the corresponding anterior part is supplied by branches from the umbilical vein (Fig. 6). Blood drained into the left branch of middle hepatic vein at and after CS18 (Fig. 5D).

### Liver segmentation

#### Lobated embryonic livers

We compared developing human livers with those of mice because this species has a lobated liver architecture with fissures to define units (arrows in Fig. 6A). At ED14 (comparable to CS18), mouse livers contained two subdiaphragmatic dorsolateral lobes, a single ventromedial lobe with left and right halves at each side of the gallbladder, and free caudate and quadrate lobes on the dorsal (posterior) side of the liver (Fig. 6B and Supplementary Figure 2). The liver hilum was positioned at the intersection between the dorsolateral and ventromedial lobes. The left and right dorsolateral lobes were drained by the left lateral hepatic vein, which was a tributary to the middle hepatic vein, and the right hepatic vein that directly entered the inferior caval vein (Fig. 6D). The middle hepatic vein drained the ventromedial lobe via a left medial hepatic tributary, a continuation of the middle hepatic vein with left and right tributaries and a right medial (accessory) hepatic vein. The caudate process, an entirely free lobe in the mouse, was drained by the caudate veins that entered the inferior caval vein (Fig. 6B). The quadrate lobe was poorly developed in the mouse embryo, so that gallbladder and umbilical vein were adjacent structures (Fig. 6A) and Cantlie’s line through the gallbladder and inferior caval vein occupied a more oblique position (Fig. 6C).

#### Human embryonic liver

In the CS18 human liver, major hepatic veins drained both dorsolateral regions of the liver (Fig. 6F). The right hepatic vein drained directly into the inferior caval vein, while the left hepatic vein drained into the middle hepatic vein. This middle hepatic vein also drained the ventromedial region of the liver (Fig. 6E) via a right medial (accessory) hepatic vein, a continuation of the middle hepatic vein with left and right tributaries, and a left medial hepatic vein. This configuration of the main tributaries was observed in all 6 embryos between 6.5 (CS18) and 10 weeks studied, albeit that course of the lateral hepatovenous tributaries of the ventromedial region could vary near the inferior caval vein by merging with the tributary from the dorsolateral region. Furthermore, a separate hepatic vein drained the caudate lobe and process (Fig. 5). These findings show that the course of the hepatic veins is, apart from the area directly surrounding the inferior caval vein, comparable in lobated and non-lobated livers, such as the human liver. A similar conclusion can be reached for the portal veins: branch #1 of the right portal vein supplies the right dorsolateral region and branches #2 and #3 the right ventromedial region (Fig. 6C–F). The numerous portal branches of the umbilical vein branch off in a spiraling fashion, with a generally anterior direction when passing the ventromedial region of the liver to a lateral direction when close to the dorsolateral region (Fig. 6C–F). Importantly, we did not observe the anticipated hierarchical binary division of the portal veins that underlies the segmental concept of the liver architecture.

## Discussion

Branches of the portal vein became identifiable at CS14 (~34 days), while the hepatic veins formed 5 days later (CS16 or ~39 days). The branching pattern of the portal veins in the right and left hemi-livers was initially similar. However, after the appearance of the hepatic veins and, hence, presumably after the development of a higher blood flow rate through the liver periphery, three main portal branches emerged in the right hemi-liver, whereas the original branching pattern, with >10 portal veins branching from the intrahepatic portion of the umbilical vein, was preserved in the left hemi-liver. This configuration can also be recognized in the drawing of the liver vessels of a CS19 embryo by Lassau^23^ and is identical to the configuration described by Fasel for adult human livers^15^. Similarly, by CS17 or CS18 the hepatic veins had established a configuration that was identical to that shown earlier for adult liver^28,29^. The venous systems of the liver have, therefore, acquired the adult configuration at 6.5 weeks of development (CS18).

### The branching pattern of the portal veins in the right and left hemi-liver

As a consequence of the appearance of the large intrahepatic left-right shunts and hepatic veins, the afferent blood supply to the liver via the intrahepatic portion of the umbilical vein came to lie upstream of the very limited blood supply via the portal vein (at 8 weeks, the diameter of the extrahepatic portion of the portal vein amounted to only 10–15% of that of the umbilical vein). The branching pattern of the umbilical vein was highly asymmetric, with a large continuing stem and relatively small portal branches forking off serially (“monopodial” branching pattern), whereas the branching pattern of the portal vein in the right hemi-liver was more symmetric. The highly asymmetric branching pattern of the intrahepatic portion of the umbilical vein, with large branching angles and small diameters of the portal branches, resembled that of a transport (“distributing”) vessel, whereas the more symmetric branching of the portal vein in the right hemi-liver better resembled that of tissue-supplying (“delivering”) vessels^30^. A “distributing” configuration appears to minimize pumping power (power-cost optimization) and seize of the vascular tree (material-cost optimization)^31^. A large difference in diameter and length of daughter branches in non-pulsatile (venous) flow systems increases the efficiency of the system during growth (decreases the allometric constant)^32^. Although these findings clearly point at the importance of hemodynamics for liver development and vascular architecture, no flow measurements are available until the fetus is 19 weeks old^33^. The observed monopodial branching pattern of the umbilical vein is, nevertheless, clearly at odds with the supposedly dichotomous branching pattern of the portal vein that underlies Couinaud’s segmental liver anatomy.

### The branching pattern of the hepatic veins

Like most studies^34^, we observed 2 or 3 main hepatic-vein outlets into the inferior caval vein. If only 2 stems were present, the third vein merged with the middle hepatic vein near its exit from the liver. More peripherally, we and others^8,35^ observed a more constant distribution of the hepatic veins, with 5 or 6 major tributaries draining Couinaud’s segments ##2–7. The “extra” peripheral hepatovenous tributaries also merged with the middle or left hepatic vein near their exit into the caval vein. We hypothesize that the more pronounced variability in the topography of the hepatic veins near their exit into the caval vein compared to that in the liver periphery relates to their association in the periphery with the dorsolateral and ventromedial liver regions that we described in early liver development^25^ and that are separated by fissures in lobated livers^24^. While identifiable veins drain the dorsolateral regions, the ~4 hepatic veins that drain the large ventromedial region or lobe of the liver appear to follow a monopodial branching pattern, with the left medial and right “accessory” veins as first branches and the main axis extending anteriorly to split in left and right branches on top of the gallbladder (Fig. 4F). Based on the rather predictable branching pattern of the hepatic veins that resembles that in lobated livers, we posit that the human liver is “crypto-lobated”. Apparently, the lobar “guidance” for the course of the hepatic veins is not present in the immediate surrounding of the caval vein.

The main hepatic vein of the caudate lobe transformed into the intrahepatic portion of the inferior caval vein. This finding explains why often several relatively small hepatic veins from the caudate lobe drain directly into the caval vein^36^. It further demonstrates that the intrahepatic portion of the inferior caval vein derives from the most posterior hepatic vein. Together with the origin of the suprahepatic portion of the inferior caval vein from the right hepatocardiac channel, which is the common connection of the vitelline and umbilical veins with the venous sinus of the heart^25^, these findings definitively establish the origin of the inferior caval vein between the well-established right-sided subcardinal vein near the kidney and its entrance into the right atrium.

### The relation of liver lobes to Couinaud’s hepatic segments

The human liver is not lobated^24^. The only lobes that are identifiable are the caudate and, to a lesser extent, the quadrate lobes. However, malformed livers with identifiable lobes are sometimes seen^37^. In lobated livers, the hepatic veins are found in the center of each lobe^38^, whereas they define the peripheral boundaries of the surgical segments of the liver. This discrepancy reveals the difference between the lobar and segmental anatomy of the liver. The lobar territories can, nevertheless, be identified in the non-lobated human liver. Both dorsolateral lobes (segments #2 and #7 in Fig. 6) can be recognized in adult livers by the topography of the dorsolateral branches of the portal vein (*e*.*g*. Figures 1,2 in^12^). On the left side, this vessel is the most distal branch of the umbilical portion of the left portal vein. In this respect, Couinaud’s “unnatural” left portal fissure^39^ properly defines this boundary. The hepatic vein draining the left dorsolateral lobe is the left lateral hepatic vein. The situation in the right hemi-liver is less clear-cut, but segment #7 probably represents the right dorsolateral lobe^40^. There is, however, no landmark vessel to delimit this segment from the remaining part of Couinaud’s posterior sector. For this reason we distinguished only 7 segments in Fig. 6. Similarly, the Spigelian sub-lobe of the caudate lobe has a distinct left boundary, but the rightward boundary of the caudate process is not so apparent. If we define the caudate process by its drainage territory (Fig. 5; *cf*.^26^), it can be much larger than usually shown, does resemble Couinaud’s segment #6, and is similar to the caudate process of lobated livers (Fig. 6). In this respect it is of interest that segment #6 develops, according to Couinaud, relatively late and is most prominent in human livers^39^.

Couinaud’s segments ##3–5, and #8 correspond with the ventromedial lobe of lobated livers. The gallbladder and middle hepatic vein demarcate Cantlie’s line. Since no portal vein branches passed this line (Figs 1 and 6), it is an accepted plane to separate the ventromedial lobe into left and right halves. However, no good landmarks are available to further divide the left and right halves of this large lobe into sectors. In the American model, the umbilical vein or round ligament serves as a sector boundary to subdivide the left-sided portion of the ventromedial lobe. Because part of the umbilical vein persists as the umbilical portion of the left portal vein, it forced Couinaud to come up with a very small left lateral hepatic sector (segment #2^16^;). However, the umbilical portion of the left portal vein is special as it lies very superficial and can be dissected free^16^. It is, therefore, probably not suitable as a landmark to subdivide the left-sided part of the ventromedial lobe into segments #3 and #4 would, if it were only, because this criterion would qualitatively differ from that (the right medial (accessory) hepatic vein) used to subdivide the right-sided part of the ventromedial lobe into segments #5 and #6. As we argued above, however, these hepatic veins follow a rather predictable course in the ventromedial lobe or region, so that we can identify 4 sectors. Using the portal and hepatic veins as criteria to delineate liver regions, we can, therefore, only identify the 7 sectors described by Hjortsjö^5,8^.

In humans, the quadrate lobe develops between the gallbladder and umbilical vein^27^, but this lobe remains a rudimentary structure in mice (Fig. 6A,B), so that the umbilical vein (round ligament after birth) lies close to the gallbladder. Such a configuration is also seen in humans with a rudimentary quadrate lobe^27^.

### Is there a developmental origin of the surgical liver segments?

The discrepancy between the branching pattern of the portal vein and the distribution of hepatic segments contradicts the putative embryological origin of Couinaud’s segmental liver anatomy^39^. Instead, the striking similarity of hepatic segments based on the distribution of the portal and hepatic veins (the French model) or on the distribution of the bile ducts and hepatic arteries (the American model) is truly remarkable because of the large time window between the appearance of the liver veins and the bile ducts/arteries: the intrahepatic bile ducts start to remodel into tubular structures only after 10 weeks of development and extend only slowly into the liver periphery^41–44^. The development of the hepatic artery accompanies bile duct development^41^. The similarity of the liver segments according to the French and American models suggests, instead, that they share a functional basis, e.g. that imposed by perfusion and metabolic demands. Accordingly, a comparison of Fasel’s and our models shows that the initially similar-sized portal veins in the left hemi-liver increase differentially in size, with some becoming much larger than others. We, therefore, suggest that Couinaud’s segments represent similar sized liver units (8–15% of the total liver volume, with only segment #8 being much larger (24%^45^;) that are supplied by hepatic arteries and an adaptively enlarged subpopulation of portal vein branches. We further posit that liver segments as defined in the French and American models represent surgically removable quantities of tissue with fractal properties: a large surgical segment (e.g. #8) can be split in 2 subsegments with the same approach as used to delineate the regular surgical segments^46^.

## Conclusions

The venous systems of the liver acquire the adult configuration at 6.5 weeks of development. The topography of the portal and hepatic veins was similar to that in lobated livers, showing that the human liver is crypto-lobated. The monopodial branching pattern of the umbilical vein is not compatible with the dichotomous branching pattern of the portal vein that underlies Couinaud’s segmental liver anatomy. We, therefore, concluded that Couinaud’s model does not have an embryological origin. The similarity of the segmental boundaries of the French and American models suggests, instead, that an adaptable mechanism underlies the segmental anatomy of the liver, e.g. that imposed by perfusion and metabolic demands.

## Materials and Methods

### Ethics

This study was undertaken in accordance with the Dutch regulations for the proper use of human and animal tissue for medical research purposes. Anonymized specimens were included from the historical collections of human embryos of the Departments of Anatomy and Embryology, Leiden University Medical Center (LUMC), Leiden, and the Academic Medical Center (AMC), Amsterdam, The Netherlands, that were donated for scientific research. The Dutch collections that we cite as sources were established in the 1950s–1970s by the late professors Dankmeijer (Leiden) and Los (Amsterdam). Furthermore, we have included a historical collection of mouse embryos from the Department of Anatomy and Embryology, AMC, Amsterdam, which was established in the 1970s–1980s by the late professor Los (Amsterdam). The study of historical collections is exempt from approval by a Medical Ethics Committee in the Netherlands. In addition, human embryos from the Carnegie Collection, Washington D.C., USA were included from the Digitally Reproduced Embryonic Morphology (DREM) project (Dr John Cork; Cell Biology & Anatomy, LSU Health Sciences Center, New Orleans; https://www.ehd.org/virtual-human-embryo/about.php, http://virtualhumanembryo.lsuhsc.edu), who made digitized sections available to us.

### Embryos

We reconstructed 12 human embryos between 5 and 10 weeks of development that represented high-quality specimens of the historical collections of AMC and LUMC, which together contain over 150 human embryos. In addition, the digitized human embryos of the Carnegie Collection were used. The criteria of O’Rahilly as modified in 2010 were used to determine the Carnegie stage of development^47^. Brain development^48^ and the return of the physiological hernia between 9 and 9.5 weeks of development^49^ were used to estimate the age of the embryos after CS23. Specimens of 8–10 weeks development are also referred to as embryos. Because most mammalian species differ from human in having lobated livers with fissures that can serve as topographic landmarks, we also studied mouse embryos from the AMC collection.

### Image acquisition, 3D-reconstruction and visualization

Digitization of the stained sections, processing of the digital images, and voxel size calculation were performed as described^50^. AMIRA (version 6.2; base package; FEI Visualization Sciences Group Europe, Merignac Cedex, France) was used to generate 3D reconstructions. Delineation of the liver contour, blood vessels/networks, and structures of interest was performed manually (Supplementary Figure 1). Polygon meshes from all reconstructed materials of an embryo were smoothened (Supplementary Figure 1) and exported via ‘vrml export’ to Cinema 4D (MAXON Computer GmbH, Friedrichsdorf, Germany). The accuracy of the remodeling process was safeguarded by simultaneous visualization in Cinema 4D of the original output from Amira and the remodeled Cinema model (Supplementary Figure 1). Subsequently, the Cinema 3D-model was exported via ‘wrl export’ to Adobe portable device format (PDF) reader version 9 (http://www.adobe.com) for the generation of 3D-interactive PDF files (Supplementary Figure 2).

## Electronic supplementary material

Supplementary Figure 1

Supplementary Figure 2
