# Supplementary material for: Human liver segments: role of cryptic liver lobes and vascular physiology in the development of liver veins and left-right asymmetry
Source: Sci Rep. 2017 Dec 7;7:17109. doi: 10.1038/s41598-017-16840-1 (PMC5719430; doi:10.1038/s41598-017-16840-1)
Supplement: Supplementary file 1 — Supplementary Figure 1 [file 41598_2017_16840_MOESM1_ESM.pdf]

Human liver segments: role of cryptic liver lobes and vascular physiology in the development of liver veins and left-right asymmetry

Jill P.J.M. Hikspoors, Mathijs M.J.P. Peeters, Nutmethee Kruepunga, Hayelom K. Mekonen, Greet M.C. Mommen, S. Eleonore Köhler, Wouter H. Lamers

Supplemental Figure 1:

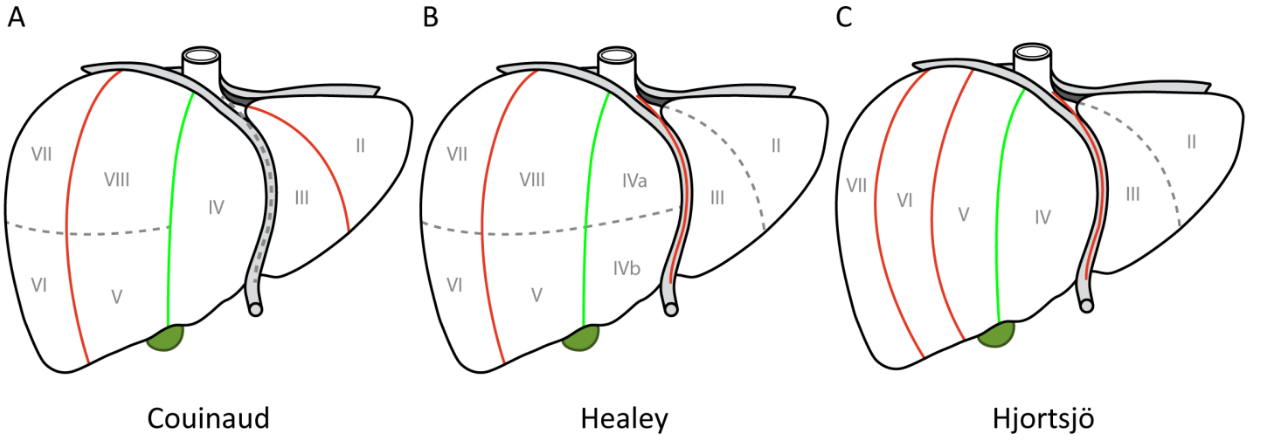

Segmentation models. Panels A-C show Couinaud's, Healey's and Hjortsjö's concepts, respectively. Cantlie's line (green) subdivides the liver into a right and left hemi-liver in all models. Whether the falciform ligament subdivides the left hemi-liver into two segments (## 3,4 in A: grey interrupted line) or sectors (## 3,4 in B,C: red line) is debated. In Healey's model the medial sector (#4) has 2 segments (#4a,b). In all models, the right hemi-liver is subdivided into an anterior and posterior sector, but Hjortsjö's model also has intermediate sector. Couinaud's and Healey's models have 4 segments in the right hemi-liver.

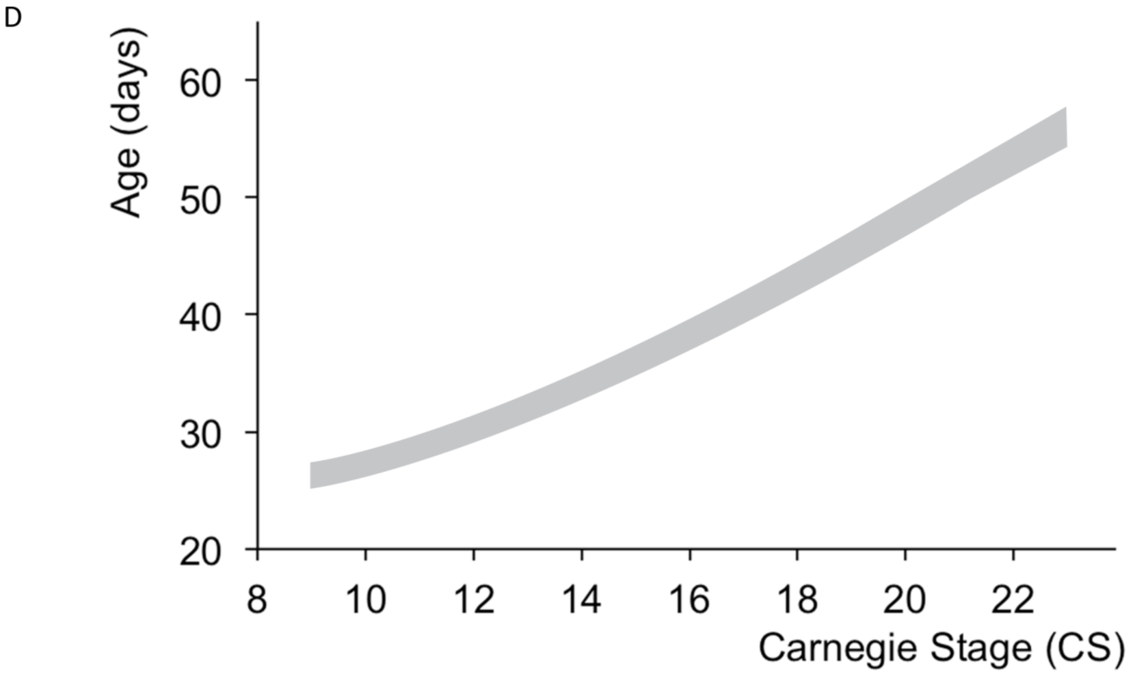

Correlation between Carnegie Stages and embryonic ages. (adapted from Table 1 (O'Rahilly et al. 2010). The lowest and highest age per Carnegie Stage is given in panel D.

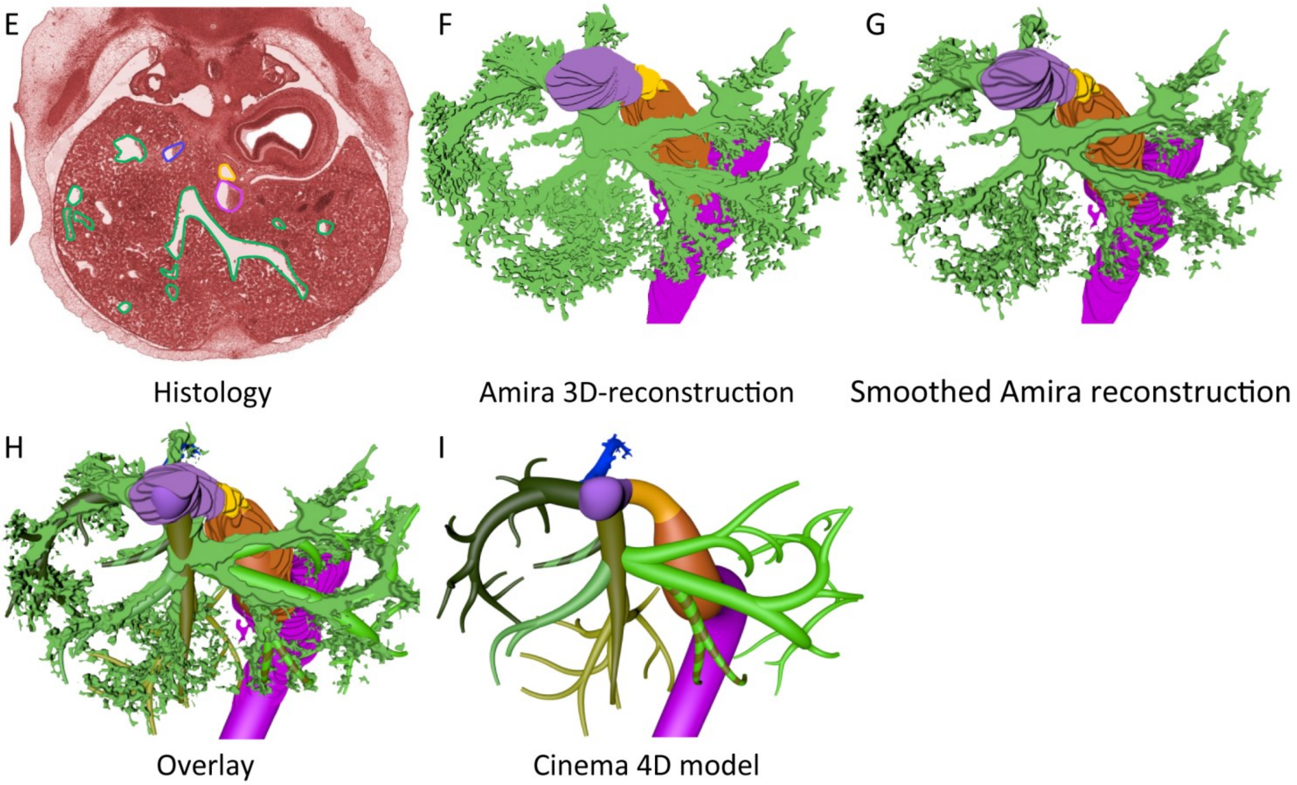

From sections via Amira 3D to Cinema 4D. The individual sections (E) used for the Amira 3D reconstruction are still visible (F). Panel G shows a smoothed visualization. Simultaneous visualization of Amira 3D and Cinema 4D, and the remodeled Cinema model are shown in panels H and I.
