# Supplementary material for: Human liver segments: role of cryptic liver lobes and vascular physiology in the development of liver veins and left-right asymmetry
Source: Sci Rep. 2017 Dec 7;7:17109. doi: 10.1038/s41598-017-16840-1 (PMC5719430; doi:10.1038/s41598-017-16840-1)

# Human liver segments: role of cryptic liver lobes and vascular physiology in the development of liver veins and left-right asymmetry

*Jill P.J.M. Hikspoors, Mathijs M.J.P. Peeters, Nutmethee Kruepunga, Hayelom K. Mekonen, Greet M.C. Mommen, S. Eleonore Köhler, Wouter H. Lamers*

**Supplemental Figure 2: Three-dimensional (3D) PDFs of human hepatic venous development at CS14, CS15, CS16, CS17, CS18, CS20 and 10 weeks. The hepatic venous development of a murine embryo at ED14 is included on the last page.**

Download the PDF-file (3D PDF can be opened on any computer as long as it contains Adobe PDF reader).

3D PDF becomes activated after 'clicking' with the mouse on the embryo. A toolbar appears at the top of the screen that includes the option 'model tree'. The model tree displays a material list of structures in the upper box and preset viewing options in the lower box. The list of visible structures can be modified by marking or unmarking a structure.

Furthermore, a structure can be rendered transparent by selecting that option from the drop-down menu after selecting the structure with the right mouse button. To manipulate the reconstruction, press the left mouse button to rotate it, the scroll button to zoom in or out, and the left and right mouse buttons simultaneously to move the embryo across the screen. The slicer button in the toolbar allows cross sections to be done. The plane of section can be adjusted with the offset and tilt options.

The color code is identical to that in the Figures and all structures are listed by name in the 'model tree'.

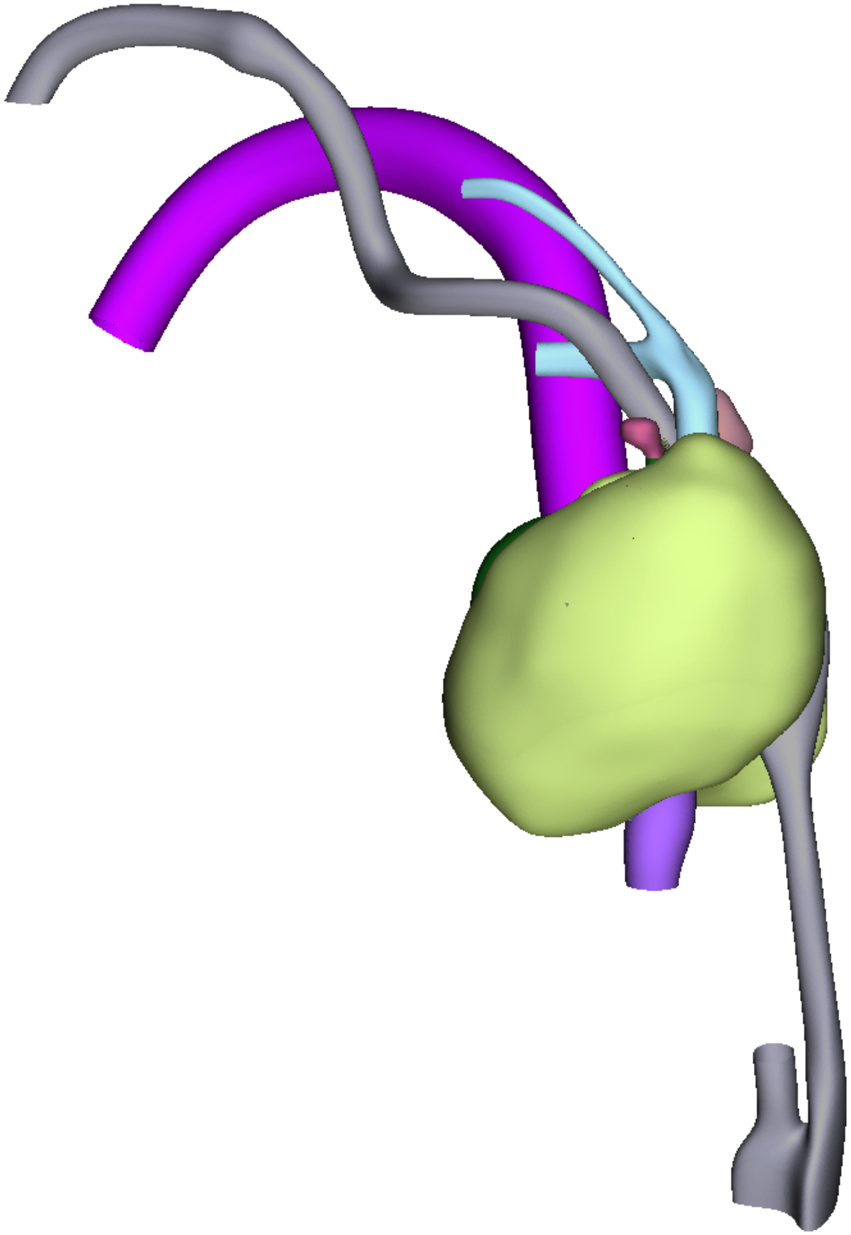

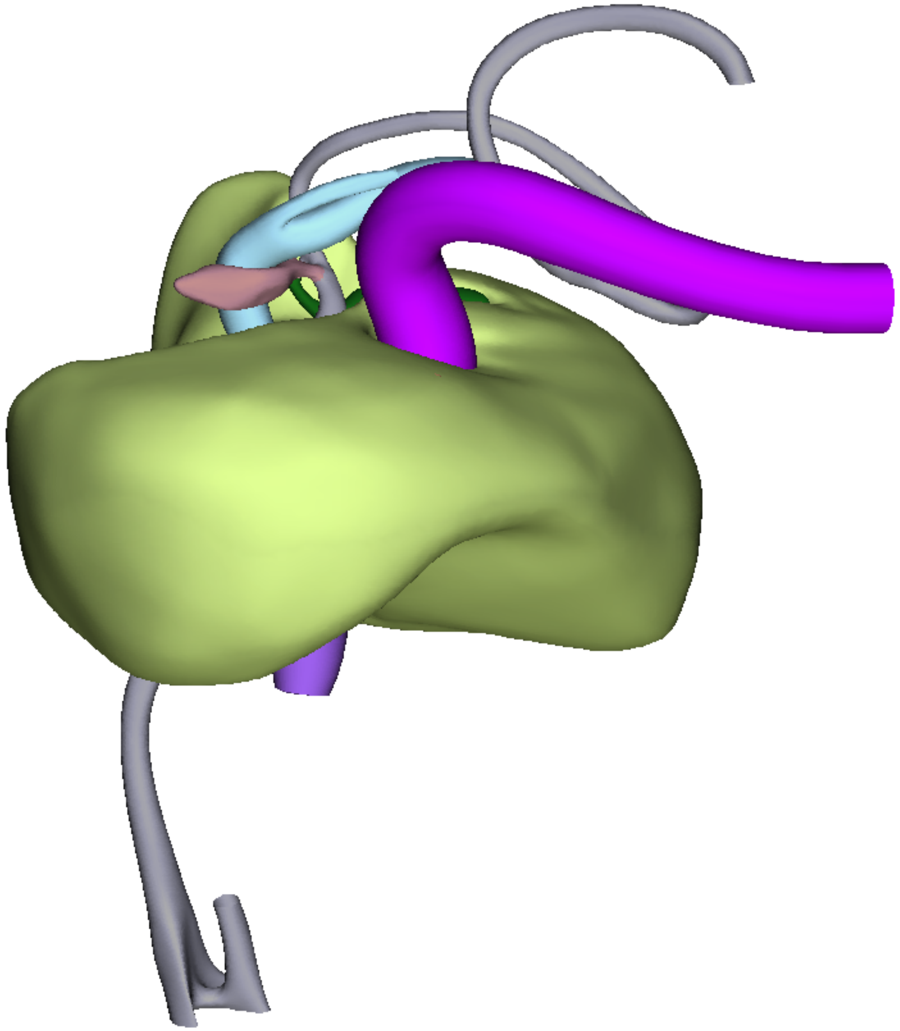

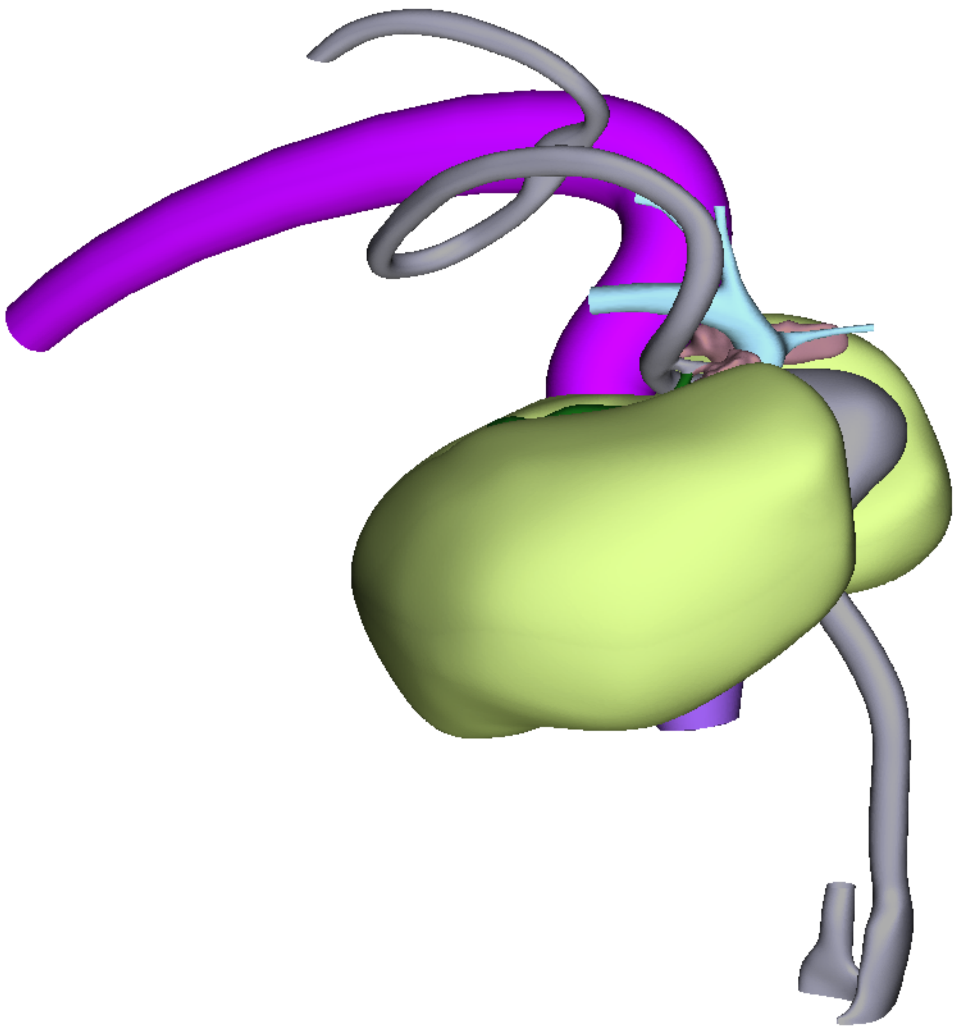

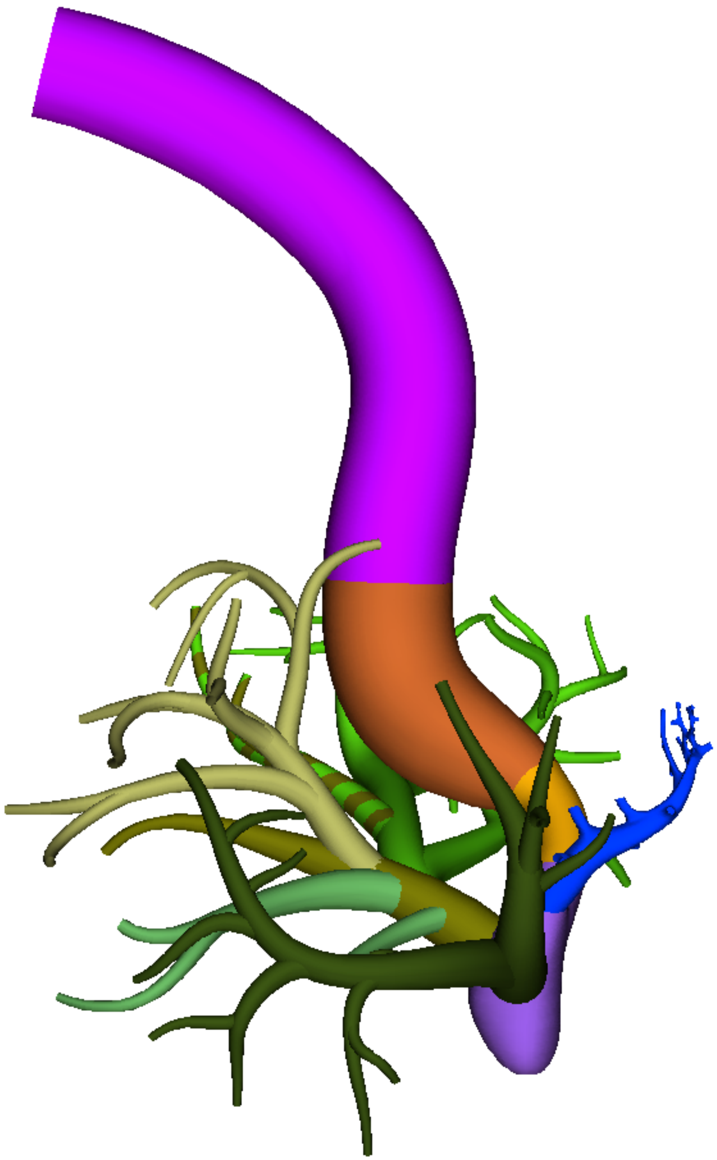

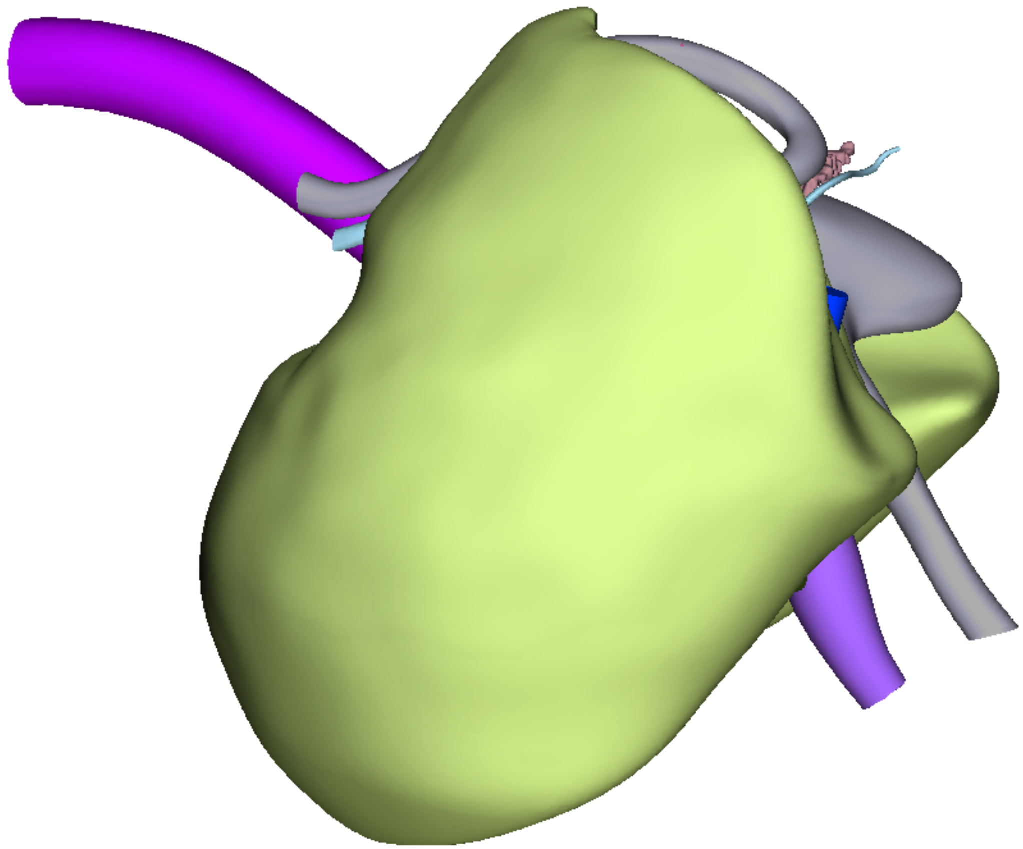

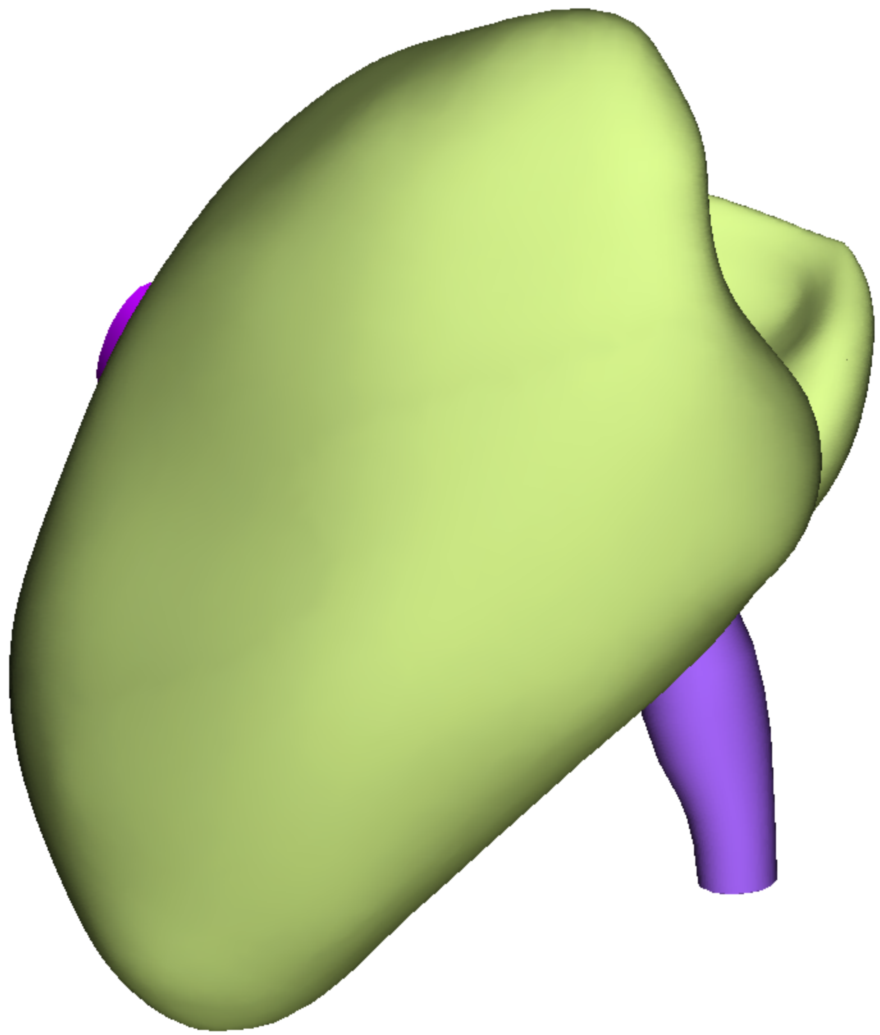

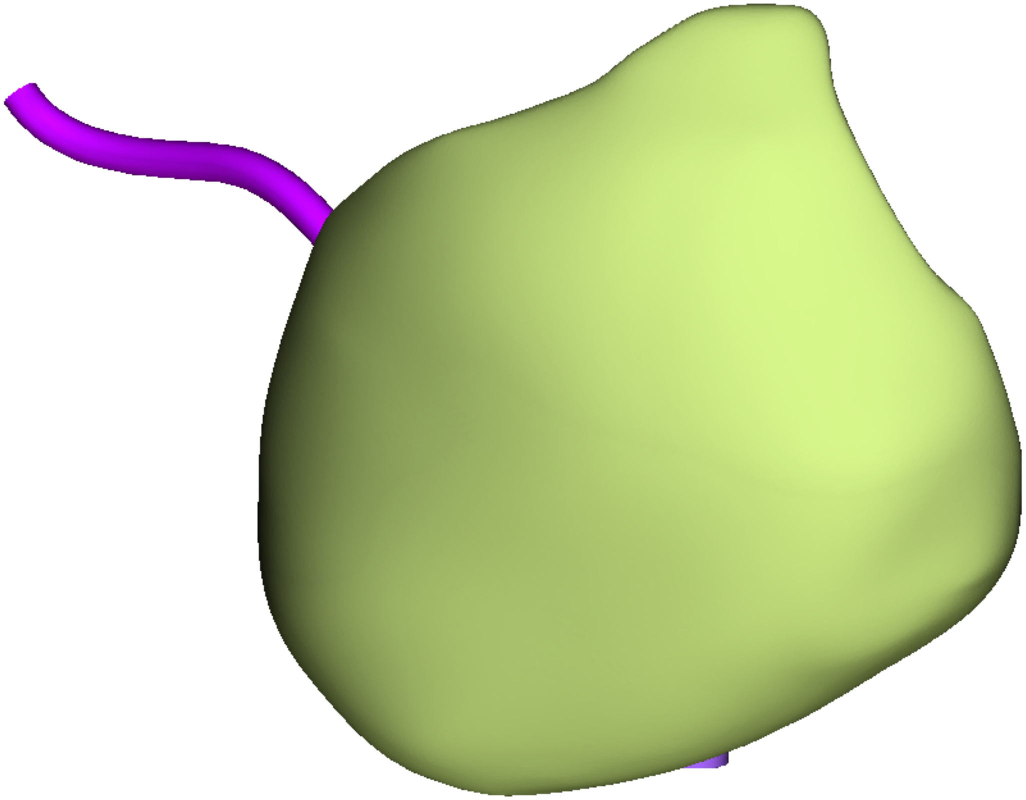

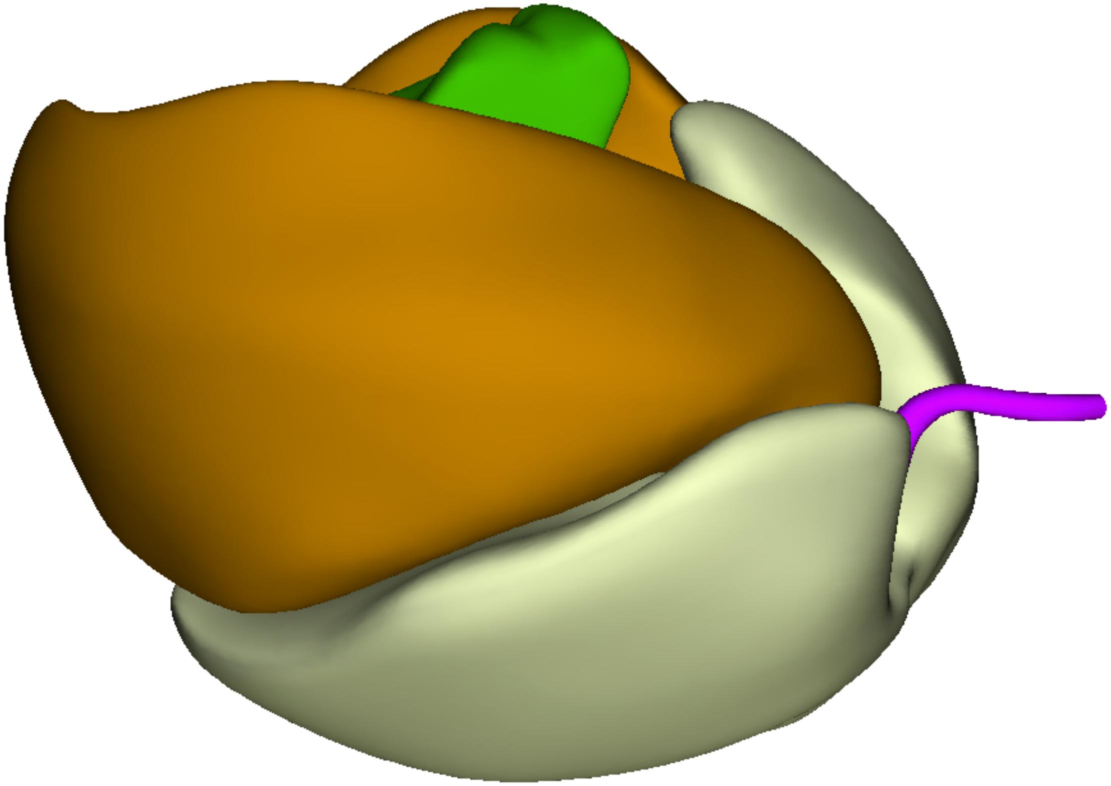

Supplement: Supplementary file 2 — Supplementary Figure 2 [file 41598_2017_16840_MOESM2_ESM.pdf]
